# Inhibitory Effects of Edaravone in ****β****-Amyloid-Induced Neurotoxicity in Rats

**DOI:** 10.1155/2014/370368

**Published:** 2014-04-02

**Authors:** Feng He, Yan-Ping Cao, Feng-Yuan Che, Lian-Hong Yang, Song-Hua Xiao, Jun Liu

**Affiliations:** ^1^Department of Neurology, Linyi People's Hospital, Linyi City, Shandong 276000, China; ^2^Department of Out-Patient, Yulin Teacher's College Hospital, Yulin, Guangxi 537000, China; ^3^Department of Neurology, Sun Yat-Sen Memorial Hospital, Sun Yat-Sen University, Guangdong 510120, China

## Abstract

Amyloid protein can damage nerve cells through a variety of biological mechanisms including oxidative stress, alterations in calcium homeostasis, and proapoptosis. Edaravone, a potent free radical scavenger possessing antioxidant effects, has been proved neuroprotective effect in stroke patients. The current study aimed to investigate the effects of EDA in an A**β**-induced rat model of AD, by studying A**β**_1–40_-induced voltage-gated calcium channel currents in hippocampal CA1 pyramidal neurons, learning and memory behavioral tests, the number of surviving cholinergic neurons in the basal forebrain, and the acetylcholine level in the hippocampus in this rat model of AD. The results showed that the A**β**_1–40_-induced increase of *I*_Ca_ can be inhibited by EDA in a dose-dependent manner. Treatment with EDA significantly improved A**β**_1–40_-induced learning and memory performance. Choline acetyltransferase positive cells in basal forebrain and acetylcholine content in the hippocampus were increased by the administration of EDA as compared with the non-EDA treated A**β**_1–40_ group. These results demonstrate that EDA can inhibit the neurotoxic effect of A**β** toxicity. Collectively, these findings suggest that EDA may serve as a potential complemental treatment strategy for AD.

## 1. Introduction

Alzheimer's disease (AD) is a neurodegenerative disease, whose pathogenesis is not fully understood. One factor known to promote the occurrence of AD is oxidative stress. Edaravone (EDA) is a free radical scavenger, which can reduce the damage caused by oxidative stress. In Japan, EDA has been widely used in the treatment of ischemic cerebrovascular disease since 2001. Recent experimental and clinical studies have revealed that EDA is also capable of producing neuroprotective effects in spinal cord infarction [1], multiple sclerosis [2], cerebral hemorrhage [3], aneurysms [4], brain injury [5–7], amyotrophic lateral sclerosis (ALS) [8, 9], and Parkinson's disease [10, 11]. An effect of EDA in the treatment of AD has also been reported [12] and results from a small sample clinical trial showed improved cognitive performance and daily living activities in patients with mild-to-moderate AD in China.

A*β*, a neurotoxic substance, exerts complex biological effects. It damages nerve cells by oxidative stress, alters calcium homeostasis, and activates a variety of proapoptotic pathways, all of which play an important role in the pathogenesis of AD. Therefore, an important area of AD research involves that of identifying agents capable of inhibiting A*β* neurotoxicity. It has been reported that EDA reduced the oxidative damage caused by A*β*_25–35_ in PC12 cells, decreased apoptosis, and increased intracellular glutathione and superoxide dismutase concentrations [13]. Additional mechanisms through which EDA can reduce the generation of A*β* toxicity involve interfering with hydrolysis sites of the amyloid precursor protein (APP), increasing the activity of *α*-secretase, and reducing the hydrolysis of *β*-secretase upon APP [14]. Within our laboratory we showed that EDA inhibits A*β*_1–40_-induced VGCC current enhancement using a patch clamp technique [15]. In the present study, we expand upon these findings and apply EDA and A*β*_1–40_ together to observe the changes in VGCC currents of rat hippocampal pyramidal cells, as well as learning and memory responses in a rat model of AD, and assess survival of cholinergic neurons and levels of acetylcholine in specific brain areas. Therefore, this study provides important new data critical for understanding the neuroprotective effects and mechanisms of EDA against A*β* toxicity.

## 2. Material and Methods

All experiments were carried out in accordance with guidelines approved by ethical committee of Sun Yat-Sen University, which includes minimizing the number of animals used and their suffering.

### 2.1. Electrophysiology Experiments

#### 2.1.1. Slice Preparation

Standard techniques were used to prepare 310 *μ*m thick acute hippocampal slices from 14–21-day-old SD rats (provided by Guangdong Medical Laboratory Animal Center). Briefly, rats were anesthetized with sodium pentobarbital and decapitated. Brains were quickly removed and within 30 s placed into an ice-cold oxygenated artificial cerebral spinal fluid (ACSF) medium containing (in mmol/L) 126 NaCl, 3.5 KCl, 1.3 MgCl_2_, 2 CaCl_2_, 1.2 NaH_2_PO_4_, 25 NaHCO_3_, and 11 glucose. The ventromedial brain hemisphere was dissected to isolate and remove the rostral and caudal tips of the hippocampus. The brain tissue was mounted onto a block and transferred to the sectioning stage of a Vibratome (DTK-1000, DSK, Kyoto, Japan) filled with ice-cold ACSF such that the cutting stage and blade were completely submerged. The brain tissue was chopped into 310 *μ*m thick sections and coronal slices were placed onto nylon mesh immersed in oxygenated ACSF at room temperature (23–25°C). Recordings were conducted after at least a 1 h recovery period and within approximately 6 h of duration of the preparation.

#### 2.1.2. Electrophysiological Recordings

Slices were superfused at room temperature with oxygenated ACSF. Patch pipettes with a resistance of 4–7 MΩ when filled with the pipette solution were prepared from glass capillary tubes (B150-86-10, Sutter Company, USA) by a four-stage horizontal puller (MODEL P-97, USA). Slice patch clamp whole-cell recording experiments were performed at room temperature (23–25°C) using an EPC-10 amplifier (HEKA Company, Germany) driven by Pulse + Pulsefit software (HEKA Company, Germany). In voltage-clamp experiments, *I*_Ca_ was elicited by depolarizing to +20 mV (100 ms) from a holding potential of −90 mV. The amplitude of *I*_Ca_ was measured as the difference between the instantaneous current at the initial and maximal activating current values.

#### 2.1.3. Solutions and Drugs

The pipette solution was composed of (in mmol/L) 120 potassium gluconate, 2 NaCl, 2 MgATP, 0.3 Na_2_GTP, 1 EGTA, and 10 HEPES. The pH was adjusted to 7.2–7.3 with KOH (1 mol/L), and osmolarity was adjusted to ~285 mOsm with KCl (2 mol/L). A*β*_1–40_ was stored as stock solutions (100 *μ*mol/L) in H_2_O at −20°C and was maintained for at least 7 d at 37°C before use to allow the formation of fibril aggregation. A*β*_1–40_ was diluted in ACSF to a concentration of 1 *μ*mol/L before use. EDA (MCI-186), purchased from BIOMOL, was dissolved in ACSF in concentrations of 1, 10, 100, and 300 *μ*mol/L. CdCl_2_ were dissolved in ASCF with a final concentration of 150 *μ*mol/L.

#### 2.1.4. Data Analysis

Currents were normalized to membrane capacitance to calculate current densities (pA/pF). Cell membrane capacitance was determined online using the Pulse + Pulsefit software program. Graphics and statistical data analysis were performed using EXCEL 2007. Results are presented as means ± SEM. Statistical analysis was performed using paired* t*-tests and one-way ANOVA. A value of *P* < 0.05 was required for results to be considered statistically significant.

### 2.2. AD Animal Models

Adult male SD rats (*N* = 48), weighing 250–300 g, were provided by the Experimental Animal Center at Sun Yat-Sen University. Rats were randomly divided into three groups consisting of sham, A*β*, and EDA-A*β*, with 16 rats in each group. To generate the AD animal model, A*β*_1–40_ was diluted to 1 *μ*g/*μ*L with sterile saline and incubated for one week at 37°C. Rats were anesthetized with 10% chloral hydrate (300 mg/kg) injected intraperitoneally. Rats were then fixed onto the stereotaxic apparatus, hair was cut, and skin was disinfected. A longitudinal incision was made along the midline to expose bregma. Microinjections were performed bilaterally into the hippocampus using the coordinates of AP-3.0 mm, L 2.0 mm, V 2.9 mm (bregma as the “zero point”). Condensed A*β*_1–40_ (10 *μ*L) was bilaterally injected slowly into each hippocampus in the A*β* and A*β*-EDA groups. The same volume of saline was injected in the sham group. The injection time was 5 min and the needle was maintained at the injection site for 2 min before the needle was slowly withdrawn.

### 2.3. Drug Treatment

EDA was injected intraperitoneally in the A*β*-EDA group at a dose of 0.3 mg/kg, twice daily for 2 weeks. Saline was injected in the A*β* and the sham group, twice daily for 2 weeks.

### 2.4. Behavior Test

All rats were subjected to the Morris water maze test at five weeks after surgery. Testing was conducted over a 5-day period. The initial test procedures involved a navigation test, also known as acquired training. Rats were trained 4 times in the morning and 4 times in the afternoon each day with each training interval being 15–20 min. The quadrant designated for initial placement of the rat was randomly selected. Rats were placed in the water facing the wall. If the rat climbed onto the platform and remained a minimum of 3 seconds after swimming for some time, this was considered a successful trial and the time spent locating the platform (the escape latency) was recorded. If the rat failed to locate the platform within 120 s, it was directed to the platform where it was permitted to remain for a 10-second interval and an escape latency of 120 seconds was assigned. Escape latencies were recorded for each animal during this initial 5-day training period. The second phase of testing consisted of the space exploration test, also known as exploratory training. On the day following acquiring training, the platform was removed to conduct the exploratory training which lasted 120 s. The quadrant containing the original platform was considered the target quadrant. Rats were placed in the water facing the wall in the quadrant opposite to the target quadrant. The amount of time in the target quadrant and number of passes through the original platform location (piercing times) were recorded during the 120 s test period.

### 2.5. ChAT-Positive Cells in the Basal Forebrain Stained by Immunohistochemistry

Upon completion of behavioral testing, rats were perfused with 4% paraformaldehyde; the brain was removed and postfixed in the 4% paraformaldehyde for 6–12 hours at 4°C. The brains were maintained in sucrose solution 1 (50 g/L in PBS) for 8 h and in sucrose solution 2 (300 g/L in PBS) for 8 h. The basal forebrain was dissected, dehydrated, transparent, and embedded in the paraffin following the directions included in the kit. Coronal sections of 5 *μ*m thickness were cut on a cryotome. Every fourth slice was collected for a total of 10 slices from each basal forebrain sample. ChAT immunohistochemistry was performed according to the directions provided in the kit. Three slices containing the medial septal nucleus (MS) and vertical arm nucleus of the diagonal band (VDB) were used for cell counting. The number of positive cells was counted within 5 randomly selected and nonoverlapping areas (40 × 10) of each slice. Cells that were not completely located within the field of view were not included in the total cell counts. The average number of cell counts from each animal was then used in the analysis.

### 2.6. ACh Content Analyzed by High Performance Liquid Chromatography (HPLC)

The hippocampus was quickly dissected on ice, weighed, and stored in the liquid nitrogen. Tissues were then transferred to an EP tube containing perchloric acid (0.1 mol/L, 1 mL/0.1 g of brain tissue) with 0.04% (w/v) Na_2_S_2_O_5_ and 0.04% (w/v) EDTA. Tissues were homogenized using an ultrasonic homogenizer for 20 s and centrifuged at 14,000 r/min × 20 min at 4°C. The supernatant was filtered through a 0.2 *μ*m membrane and stored at −80°C for further analysis. The mobile phase was 0.2 mol/L Tris-maleate buffer (PH 7.0), containing TMACl 150 mg/L and OSA 10 mg/L. The flow rate was 1.3 mL/min. After separation with HPLC, the enzyme column (AChE 125U, ChO 75U) was used for postcolumn reaction, followed by electrochemical detection at 0.5 V.

### 2.7. Statistical Analysis

Data were expressed as mean ± SD. One-way ANOVA with the post hoc* Q* test or* t*-test was used for statistical analysis with the SPSS11.0 software program. *P* < 0.05 was required for results to be considered statistically significant.

## 3. Results

### 3.1. A*β*_1–40_ Increased the Amplitude of *I*_Ca_ ****in Hippocampal CA1 Pyramidal Neurons

**Figure 1:**
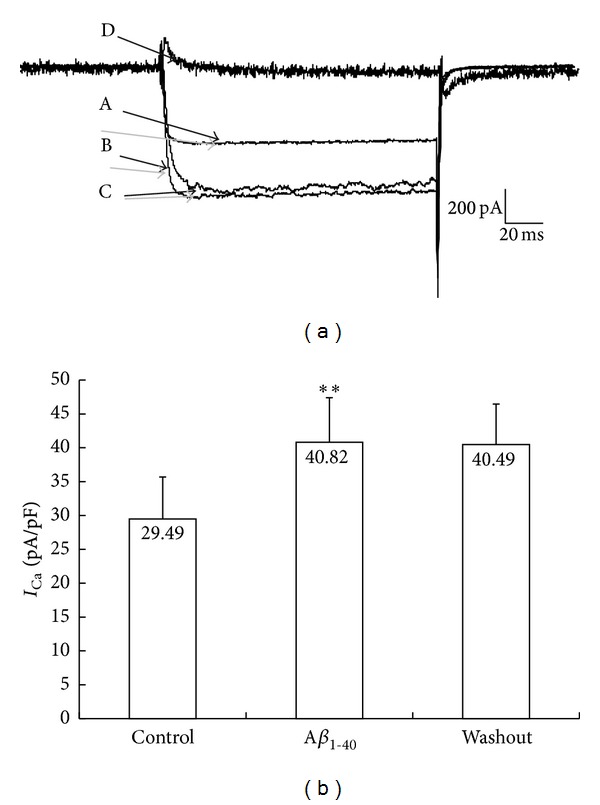
Effect of A*β*_1–40_ on *I*_Ca_ in hippocampal neuron. (a) Increase in the amplitude of *I*_Ca_ by A*β*_1–40_ (1 *μ*mol/L) in hippocampal neurons. A, control; B, A*β*_1–40_ application; C, washout; D, CdCl_2_ (150 *μ*mol/L) application. (b) Summary of data showing increases of current density (*I*_Ca_ (pA/pF)) by A*β*_1–40_. **P < 0.01 versus control.

Bath application of A*β*_1–40_ at a concentration of 1 *μ*mol/L increased the amplitude of *I*_Ca_ by 40.44 ± 12.56%; *n* = 18, *P* < 0.01, and this enhancement of *I*_Ca_ was not reversed upon A*β*_1–40_ washout (Figure 1). CdCl_2_ (150 *μ*mol/L in ACSF) blocked the inward current.

### 3.2. EDA Reduced the Amplitude of *I*_Ca_ Induced by A*β*_1–40_

**Figure 2:**
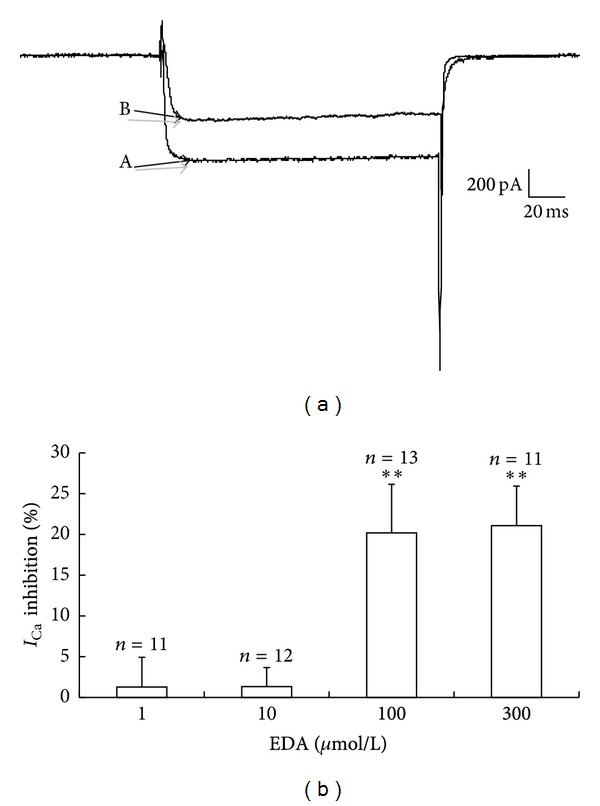
Inhibition of the action of A*β*_1–40_ by EDA in hippocampal neurons. (a) EDA reduced the amplitude of *I*_Ca_ induced by A*β*_1–40_. a, A*β*_1–40_; B, EDA (100 *μ*mol/L). (b) Summary of inhibition (percent) of *I*_Ca_ by EDA at concentrations of 1, 10, 100, and 300 *μ*mol/L. **P < 0.01 versus control.

Following the increase of *I*_Ca_ by A*β*_1–40_, EDA was applied extracellularly at concentrations of 1, 10, 100, or 300 *μ*mol/L. EDA reduced *I*_Ca_ by 1.71 ± 3.81%; *n* = 11, *P* > 0.05, 1.26 ± 2.20%; *n* = 12, *P* > 0.05, 20.18 ± 5.95%; *n* = 13, *P* < 0.01, and 21.07 ± 4.84%; *n* = 11, *P* < 0.01, respectively (Figure 2). No differences in inhibiting increased *I*_Ca_ by A*β*_1–40_ (*n* = 8, *P* > 0.05) were obtained between the 100 and 300 *μ*mol/L concentrations of EDA. No statistically significant differences were obtained among the different concentrations of EDA upon basal *I*_Ca_ of hippocampal CA1 pyramidal neurons 1.69 ± 3.27%; *n* = 9, *P* > 0.05, 2.44 ± 4.30%; *n* = 10, *P* > 0.05, 3.26 ± 4.55%; *n* = 9, *P* > 0.05, and 3.87 ± 5.58%; *n* = 18, *P* > 0.05, respectively.

### 3.3. Improved Learning and Memory Function in EDA-Treated Rats

As summarized in Table 1, rats in the A*β* group showed statistically increased latencies as compared to that in sham-operated rats (56.7 versus 20.5 s, *P* < 0.01). The average latency for rats in the A*β*-EDA group was significantly decreased (38.1 s) as compared with the A*β* group but remained significantly increased when compared to the sham group (*P* < 0.05). In the space exploration test, rats in the sham group displayed the longest residence times in the target quadrant and the maximum piercings times while rats in the A*β* group demonstrated the shortest residence times in the target quadrant and the lowest number of piercings. These differences between the sham and A*β* groups were statistically significant (*P* < 0.01). The average residence times in the target quadrant, as well as the number of piercings for rats in the A*β*-EDA group, were significantly increased as compared with that of rats in the A*β* group (*P* < 0.05).

### 3.4. ChAT Staining in Basal Forebrain by Immunohistochemistry 

**Figure 3:**
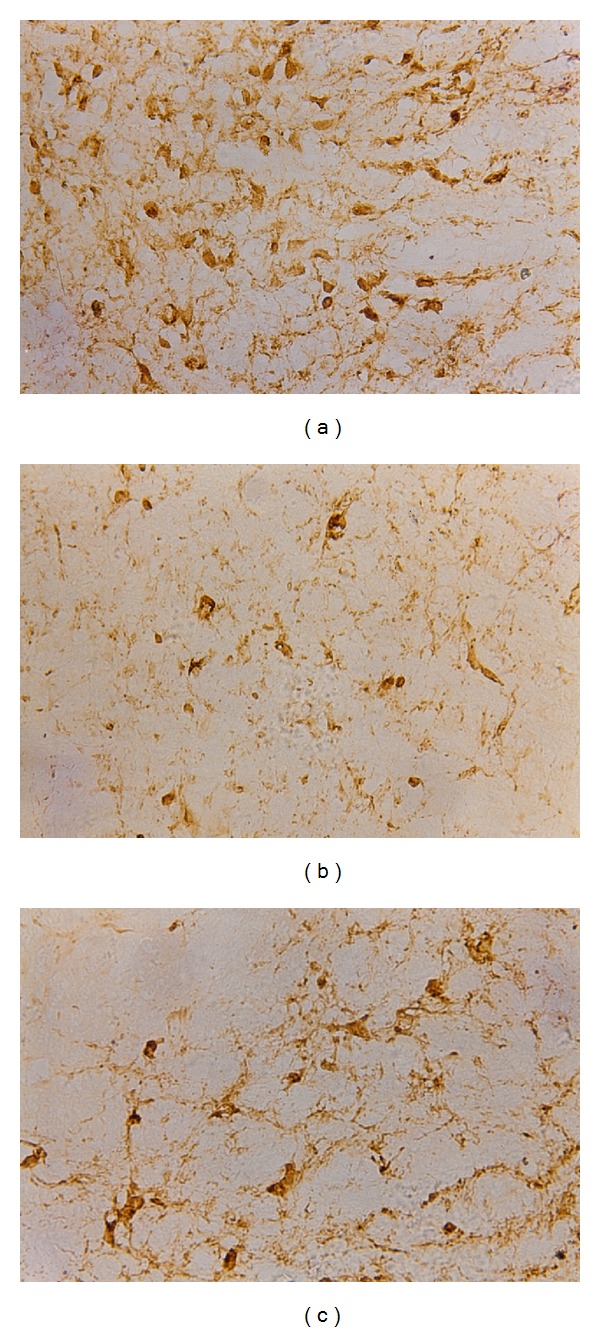
Representative stains of ChAT-immunoreactive neurons of the rats in the three groups: (A) sham; (B) A*β*; (C) EDA. The toxic effects of A*β* resulted in a significant reduction in ChAT-positive cells (b). EDA treatment significantly restored ChAT-positive cell numbers (c), but this increase remained significantly lower than the cell counts in sham group (a).

**Figure 4:**
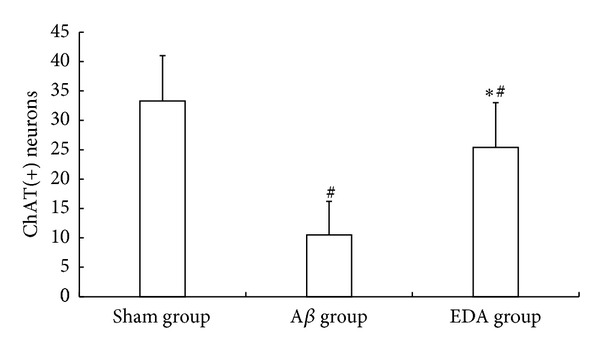
Number of ChAT-immunoreactive neurons in the three groups. *P < 0.01, compared with A*β*; ^#^ *P* < 0.01, compared with sham.

Results from immunohistochemistry staining showed that fewer ChAT-positive cells were present in the basal forebrain of the A*β* group (Figure 3(b)). After EDA treatment, ChAT-positive cell numbers were significantly increased (Figure 3(c)). Rats in the sham group displayed the highest number of ChAT-positive cells (Figure 3(a)). Cell counts as performed under high power field showed that the mean + SD values of ChAT-positive cells in sham, A*β*, A*β*-EDA groups were 33.3 ± 7.7, 10.5 ± 5.7, and 25.4 ± 7.6, respectively (Figure 4). The differences among all three groups were statistically significant (*P* < 0.01).

### 3.5. Ach Content in Hippocampus

**Figure 5:**
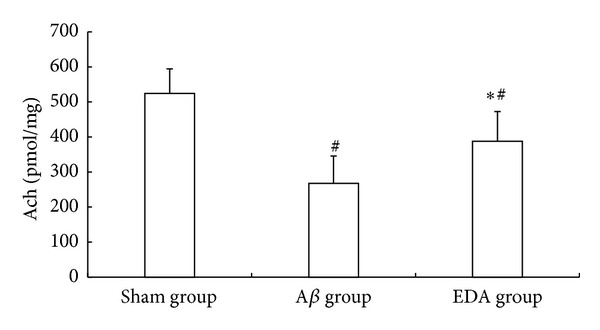
Content of Ach in the hippocampus in the three groups. *P < 0.01, compared with A*β*; ^#^ *P* < 0.01, compared with sham.

The highest content of Ach in the hippocampus was obtained in the sham group (524.3 ± 70.2 pmol/mg) and lowest in the A*β* group (267.6 ± 78.1 pmol/mg) (Figure 5). Ach content in the hippocampus of the A*β*-EDA group (387.5 ± 85.2 pmol/mg) was significantly increased as compared with that of the A*β* group (*P* < 0.01) but remained significantly lower than that in the sham group (*P* < 0.01).

## 4. Discussion

A*β*, a neurotoxic substance, exerts complex biological effects. It damages nerve cells by oxidative stress, alters calcium homeostasis, and activates a variety of proapoptotic pathways, all of which play an important role in the pathogenesis of AD [12, 16]. Due to the high amount of oxygen consumption, high levels of unsaturated fatty acids, and the relative lack of antioxidant enzymes in the brain, nerve cells are particularly vulnerable to free radicals generated by oxidative stress, which results in lipid peroxidation and the damages membrane structure. It is known that A*β* deposition activates microglia and astrocytes, which release various anti-inflammatory cytokines and free radicals such as IL-*α*, S100*β*, and TNF-*α*. A*β* can promote the generation of reactive oxygen species (ROS), which play a critical role in oxidative damage to neurons. A*β* can also activate the complement system and promote the formation of immune complexes, which attack the neurons that are malnutritioned or damaged resulting in decomposition of the cell membrane and cell death. Moreover, A*β* can directly generate free radicals or bind with metal ions (Fe^3+^, Cu^2+^) to generate OH^•^ from H_2_O_2_ or other ROS via the Fenton reaction [17]. It has been reported that free radicals can cause increases in VGCC-mediated calcium currents [18, 19], which suggests that the free radicals generated by A*β* may be involved in this effect. Data from other studies indicate that A*β* may act directly on VGCC, which can temporally increase the inward calcium current [20–22]. In the current study, A*β*_1–40_ was added to the perfusate, as a means to assess its acute effects on brain slices. With this approach, we found an instant increase in VGCC currents in hippocampal neurons, which is consistent with the findings of an earlier report by Chen et al. [20]. Of greater significance were our results demonstrating that acute EDA treatment effectively inhibited this A*β* effect in a concentration-dependent manner. Such results suggest that EDA administration can partially reverse A*β* effects upon current regulation, thereby reducing calcium influx and avoiding calcium overload. Such an effect may then represent one of the mechanisms underlying the therapeutic effects of EDA.

In our animal study, A*β*_1–40_ injected directly into the hippocampus resulted in a decrease in learning and memory function in the rats, thereby generating an AD animal model of AD. With EDA treatment of these AD rats, learning and memory scores were significantly improved. Immunostaining with ChAT revealed that cholinergic neuronal survival was higher in the basal forebrain and Ach concentration in the hippocampus was significantly higher in EDA-treated rats. These results suggest that EDA has the capacity of reducing the toxic effects of A*β*. Our findings are in accord with a number of other studies demonstrating a neuroprotective role for EDA. For example, EDA reduces A*β* expression [14], inhibits the production of inflammatory mediators leukotrienes, thus reducing the concentration of hydroxyl radical, decreases the production of the toxic carbonyl compound (carbonyl protein, advanced glycation end products, and malondialdehyde) [23], increases intracellular glutathione and superoxide dismutase concentrations [13], and reduces the expression of tau protein and glial fibrillary acidic protein (GFAP) in the cortex and hippocampus of the vascular dementia rat [24], thus reducing the highly phosphorylated tau protein concentrations [25]. In addition, EDA can stabilize the mitochondrial membrane potential, inhibit the mitochondrial-dependent apoptotic pathways, and inhibit the release of cytochrome C and the activation of Caspase-3 by reducing the Bax/Bcl-2 ratio [26]. Edaravone can also protect HT22 neurons from H_2_O_2_-induced apoptosis by inhibiting the MAPK signaling pathway moreover [27]. Taken together, the findings of these studies combined with the results of the current experiments suggest that EDA may serve as a neuroprotectant that may prove to be a particularly effective drug therapy for AD.

## 5. Conclusion

The present study demonstrated that edaravone, a free radical scavenger, could inhibit the neurotoxic effect of A*β* in rats and improve learning and memory ability of model animals through attenuation of Ca^2+^ overload, protecting cholinergic neurons in the basal forebrain, and increasing acetylcholine content in the hippocampus.

## Figures and Tables

**Table 1:** Behavior test results of the rats in each group.

Group	Number	Escape latency	Aim-quadrant stay	Time of reaching target
Sham group	8	20.5 ± 7.7 (sec)	88.6 ± 17.5 (sec)	18.4 ± 7.2
A*β* group	8	56.7 ± 15.2^#^ (sec)	44.1 ± 15.3^#^ (sec)	7.5 ± 3.6^#^
EDA group	8	38.1 ± 11.0^∗Δ^ (sec)	71.9 ± 12.8^∗Δ^ (sec)	13.2 ± 5.8^⋆※^

*P < 0.01, compared with A*β* group; ^Δ^ *P* < 0.05, compared with sham group; ^#^ *P* < 0.01, compared with sham group; ^⋆^ *P* < 0.05, compared with A*β* group; ^※^ *P* > 0.05, compared with sham group.
